# Supplementary material for: Patterns of Intron Gain and Loss in Fungi
Source: PLoS Biol. 2004 Nov 30;2(12):e422. doi: 10.1371/journal.pbio.0020422 (PMC532390; doi:10.1371/journal.pbio.0020422)
Supplement: Table S1 — Also available at http://genes.mit.edu/NielsenEtAl/. (4.3 MB ZIP). [file pbio.0020422.st001.zip › NielsenEtAl/html/387.html]

AN0131.1.NCU03423.1.MG03943.1.FG01466.1


```
 CLUSTAL W (1.82) Multiple Sequence Alignments - Introns Inserted


Sequence 1: NCU03423.1	206 aa
Sequence 2: MG03943.1	205 aa
Sequence 3: FG01466.1	196 aa
Sequence 4: AN0131.1	201 aa
Alignment Length: 208 aa
Number Identitical Residues: 70 aa
Alignment Score (without introns) 4214


MG03943.1 	MRSSPLLLLASFLR-VAMAHGDHGGYGKKPEVGPDASWMDKHMAE~EHHADHWDAASFFT
NCU03423.1	MRPSTVILAAFGLTGSALAHGDHSSRSQKPIVGENASWMVKHMAE1EHHVENFDAASFFA
FG01466.1 	MRTG--IFLSVAFATTVFAH------SQKPIVDANADWMTKHMAE1EHHVQGWDADSFFT
AN0131.1  	MLITLTTALLALSGSLVNAHG-----SHSTPTDPSADWATRHMQE1EHHIDTFDAASFFT
          	*               . **.     .:.. .. .*.*  :** * *** : :** ***:

MG03943.1 	LHDFNSDGHLDESELLRTYGVFDESNKHVTKQRKDEVTKQLMDMLDTDHDGFVAHNEFLE
NCU03423.1	LHDFDADGTWEGLEILRTYGLMDDSNKGVSQPRRDEIVRDILGLIDYDRNGVITKDEFVR
FG01466.1 	LHDYNGDGWWQAAELMRTYGLFDESNKGMGDKRKEEVRDILLGLLDKDSDSSVSRKEWMD
AN0131.1  	LHDYDSSGAWTPEEVRKTYGMDDESNAGLTEERKQEALREVFTLFDPANTGFISRDNWLH
          	***::..*     *: :***: *:**  : . *::*    :: ::*    . :::.::: 

MG03943.1 	FIH-QGKTLPDLGLGPGHHGDDEYEYEIHHWEK~YHDENTKLEDLT0HPEDIKHFEHHEK
NCU03423.1	FIEVEGKTLPDMGTGPGHHGDAEYEYEIHHWEK2YHDDNTKLEDLT~HPEDIEHFKKHEE
FG01466.1 	YVN-SGKTLPDLNTGPGHHGDDEYEYEIHHWEK~YHDDNTKLEDLT~HPEDIEHFKKHDE
AN0131.1  	LVS-EGVILPDFGFGPGHHGDIEYEYEIHHFEK~YHGEDATEEELT~HPEDIEHFKRHDE
          	 :  .*  ***:. ******* ********:** **.:::. *:** *****:**::*::

MG03943.1 	MELEQEKIDKMMASSEIMLENVPSKFRRQG-
NCU03423.1	MELEEERLAQMDK-IAIIEENIPAKFRRSG-
FG01466.1 	MEDAQDRLEAMQK-LSIVEANIPQKFRRQ--
AN0131.1  	EDEAMEKLDQLER-MAIVAANIPAKFRKGRV
          	 :   :::  :     *:  *:* ***:
```
